# Apparent diffusion coefficient of normal adrenal glands*

**DOI:** 10.1590/0100-3984.2015.0045

**Published:** 2016

**Authors:** Sara Reis Teixeira, Paula Condé Lamparelli Elias, Andrea Farias de Melo Leite, Tatiane Mendes Gonçalves de Oliveira, Valdair Francisco Muglia, Jorge Elias Junior

**Affiliations:** 1 MD, PhD, Attending Physician at the Centro de Ciências das Imagens e Física Médica (CCIFM), Hospital das Clínicas da Faculdade de Medicina de Ribeirão Preto da Universidade de São Paulo (HCFMRP-USP), Ribeirão Preto, SP, Brazil; 2 MD, PhD, Endocrinology Division of the Department of Internal Medicine, Hospital das Clínicas da Faculdade de Medicina de Ribeirão Preto da Universidade de São Paulo (HCFMRP-USP), Ribeirão Preto, SP, Brazil; 3 MD, PhD, Attending Physician at the Instituto de Medicina Integral Professor Fernando Figueira de Pernambuco (IMIP), Recife, PE, Brazil; 4 MD, PhD, Associate Professor in the Radiology Division of the Department of Internal Medicine, Faculdade de Medicina de Ribeirão Preto da Universidade de São Paulo (FMRP-USP), Ribeirão Preto, SP, Brazil

**Keywords:** Adrenal glands, Diffusion magnetic resonance imaging, Magnetic resonance imaging

## Abstract

**Objective:**

To assess the feasibility and reliability of apparent diffusion coefficient (ADC) measurements of normal adrenal glands.

**Materials and methods:**

This was a retrospective study involving 32 healthy subjects, divided into two groups: prepubertal (PreP, *n* = 12), aged from 2 months to 12.5 years (4 males; 8 females); and postpubertal (PostP, *n* = 20), aged from 11.9 to 61 years (5 males; 15 females). Diffusion-weighted magnetic resonance imaging (DW-MRI) sequences were acquired at a 1.5 T scanner using *b* values of 0, 20, 500, and 1000 s/mm^2^. Two radiologists evaluated the images. ADC values were measured pixel-by-pixel on DW-MRI scans, and automatic co-registration with the ADC map was obtained.

**Results:**

Mean ADC values for the right adrenal glands were 1.44 × 10^-3^ mm^2^/s for the PreP group and 1.23 × 10^-3^ mm^2^/s for the PostP group, whereas they were 1.58 × 10^-3^ mm^2^/s and 1.32 × 10^-3^ mm^2^/s, respectively, for the left glands. ADC values were higher in the PreP group than in the PostP group (*p* < 0.05). Agreement between readers was almost perfect (intraclass correlation coefficient, 0.84-0.94; *p* < 0.05).

**Conclusion:**

Our results demonstrate the feasibility and reliability of performing DW-MRI measurements of normal adrenal glands. They could also support the feasibility of ADC measurements of small structures.

## Resumo

**Objetivo:**

Avaliar se a medida do coeficiente de difusão aparente (CDA) das glândulas suprarrenais é factível e reprodutível.

**Materiais e métodos:**

Neste estudo foram incluídos, retrospectivamente, 32 indivíduos saudáveis divididos em dois grupos: pré-púbere (PreP) (*n* = 12; 2 meses a 12,5 anos; 4 masculinos e 8 femininos) e pós-púbere (PostP) (*n* = 20; 11,9-61 anos; 5 masculinos e 15 femininos). Imagens de difusão por ressonância magnética (DWI) das glândulas suprarrenais foram realizadas em aparelho de 1,5 T utilizando-se *b* valores de 0, 20, 500 e 1000 s/mm^2^. As medidas do CDA das glândulas suprarrenais foram obtidas pixel-a-pixel por dois radiologistas após co-registro automático do mapa de CDA com DWI.

**Resultados:**

A média dos valores do CDA da glândula suprarrenal direita foi 1,44 × 10^-3^ mm^2^/s no grupo PreP e 1,23 × 10^-3^ mm^2^/s no grupo PostP, e da glândula esquerda foi 1,58 × 10^-3^ mm^2^/s e 1,32 × 10^-3^ mm^2^/s, respectivamente. Os valores de CDA foram mais altos no grupo PreP comparados aos do PostP (*p* < 0,05). A concordância interobservador foi quase perfeita (coeficiente de correlação intraclasse: 0,84-0,94; *p* < 0,05).

**Conclusão:**

Estes resultados mostram que medir o CDA das glândulas suprarrenais é factível e reprodutível. Esta técnica poderia ser utilizada para medir o CDA de estruturas pequenas.

## INTRODUCTION

Diffusion-weighted magnetic resonance imaging (DW-MRI) has increasingly become routine in whole-body MRI protocols. The DW-MRI technique has the ability to provide qualitative and quantitative information at a cellular level, based on molecular diffusion^(1)^, that partially reflects tissue cellularity and the presence of intact cellular membranes^(2)^. Technical advances in MRI, such as the development of parallel imaging, high amplitude gradients, and multichannel coils, have enabled the use of DW-MRI for abdominal studies^(3,4)^. DW-MRI can provide useful additional information for the characterization of abdominal lesions. In addition, quantitative information provided by apparent diffusion coefficient (ADC) measurements is now recognized as a potential biomarker^(5,6)^, correlating significantly with tissue cellularity, extracellular space tortuosity, and integrity of cellular membranes^(2,7)^.

Some abdominal organs have been extensively studied by DW-MRI^(8-10)^, for assessing either focal or diffuse lesions. In adrenal glands, ADC values have mainly been used for differentiating between benign and malignant lesions^(11-15)^. However, to our knowledge, there have been no studies using ADC in order to characterize normal adrenal glands.

The purpose of this study was to assess the feasibility and reproducibility of ADC measurements of the adrenal glands in subjects with no endocrine disorder or adrenal disease.

## MATERIALS AND METHODS

### Study population

The study was approved by the Institutional Review Board of the "Hospital das Clínicas da Faculdade de Medicina de Ribeirão Preto da Universidade de São Paulo", and a waiver of informed consent was granted. Between November 2011 and December 2012, 1330 subjects underwent abdominal MRI at our institution, where DW-MRI is part of the routine protocol. Patient charts were reviewed in order to exclude subjects with abnormal radiological findings (*n* = 1273). Of the remaining 57 subjects, 24 were excluded on the basis of the clinical data: for showing hormonal disturbances (*n* = 5); for having a history of neoplasia (*n* = 5); for being under suspicion of having chronic inflammatory bowel disease (*n* = 3); for being under suspicion of having storage disease (*n* = 4); and for having an above-normal body mass index for age (*n* = 7). Another subject was excluded because radiologic data were missing. Therefore, the study sample comprised 32 healthy, normal-weight subjects without hormonal disturbances, chronic diseases, or systemic diseases. The subjects were divided into two groups: prepubertal (PreP, *n* = 12), composed of infants and preadolescents (4 males and 8 females), aged from 2 months to 12.5 years (median age, 6.8 years); and postpubertal (PostP, *n* = 20), composed of adolescents and adults (5 males and 15 females), aged from 11.9 to 61 years (median age, 35.4 years). According to the Tanner classification criteria^(16,17)^, the infants and preadolescents in the PreP group were classified as stage 1, whereas the adolescents in the PostP group were classified as stages 2, 3, 4, or 5.

### Magnetic resonance imaging

MRI was performed with a 1.5 T scanner (Achieva; Koninklijke Philips N.V., Eindhoven, the Netherlands) with an anterior 16-channel, phased-array body coil. A standard protocol for abdominal imaging, including DW-MRI, was used. All exams were collected and saved in the picture and archiving communication system for posterior analysis.

Chemical shift, respiratory-gated, transverse multislice echo-planar DW-MRI was performed with fat saturation and without intravenous contrast administration. The following sequence parameters were used: diffusion gradient *b* values of 0, 20, 500, and 1000 s/mm^2^, applied in three orthogonal directions (x, y and z) and subsequently averaged to minimize the effects of diffusion anisotropy; parallel imaging reduction factor, 2; repetition time/echo time, 5128/73 ms; echo planar imaging factor, 69; slice thickness, 5 mm; interslice gap, 0-1 mm; matrix size, 172 × 133; field of view, 345 × 321 mm; and number of excitations, 2. The whole sequence consisted of 20-25 slices, with an average acquisition time of 195 s. ADC maps were automatically generated by the MRI system, with a multiexponential model. Calculated ADC values are expressed in square millimeters per second (× 10^-3^ mm^2^/s).

### Image analysis

Images of all subjects were evaluated independently and retrospectively on a workstation by two different radiologists who were blinded to the groups. The radiologists had 3 and 5 years of experience in abdominal imaging, respectively, the latter having an additional 2 years of experience in pediatric imaging. To avoid any learning bias, the images were reviewed in a randomized fashion.

DW-MRI and ADC maps were converted into MINC format and analyzed using a free software package (Display version 1.4.2; David McDonald, Brain Imaging Centre of the Montreal Neurological Institute, Montreal, Canada).

**Figure 1:**
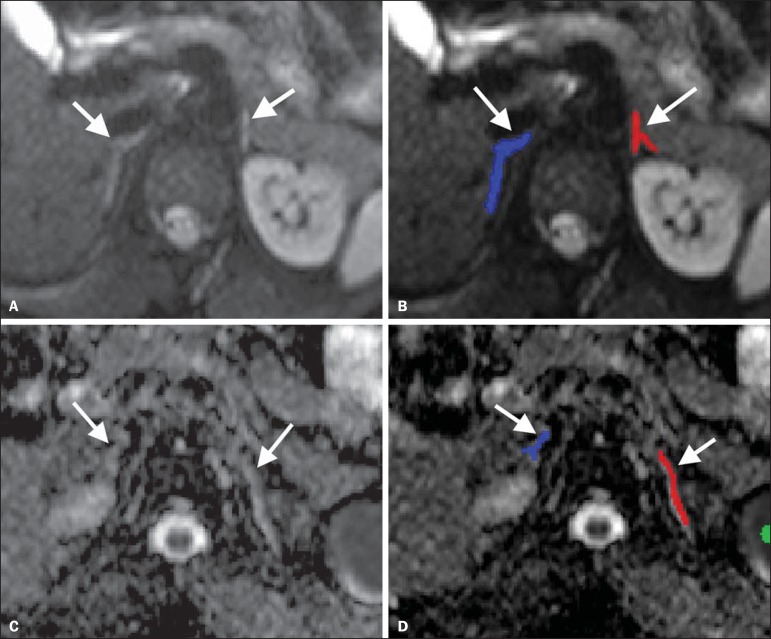
Segmentation of the adrenal glands. Example of how to segment the adrenal glands and co-register DW-MRI with the ADC map. Axial DW-MRI, *b* = 500 s/mm^2^ without labels (**A**) and with labels (**B**) on the adrenal glands. Axial ADC maps without labels (**C**) and with labels (**D**) on the adrenal glands. After thresholds have been set, the adrenal glands (arrows) are segmented manually. The segmented labels are then co-registered with the ADC map. The software automatically calculates the mean ADC values of the glands based on the regions labeled.

ADC measurements were performed on a pixel-by-pixel basis, by manual segmentation of the adrenal glands on the original DW-MRI, at a *b* value of 500 s/mm^2^. Because the adrenal glands are often irregular in shape, threshold values were established to exclude adjacent fat from the region of interest (ROI). By summing and then dividing by two the values of the voxel with the highest value closest to the adrenal gland and the voxel with the lowest value within the adrenal gland, we defined the threshold for the maximum value of the voxels of the ROIs. The ROI was then defined by selecting the outermost surrounding voxels with the same signal level of the thresholds in each slice and by further selecting the interior of the surrounded area. After adrenal gland segmentation, images were fused with the ADC map using a script. By this method, the ADC value of the ROI is automatically calculated as the average of the pixels manually chosen in each slice (Figure 1).

The margins of the adrenal glands were clearly defined on DW-MRI for all subjects. Reviewers were blinded to the age, sex, and pubertal stage of the subjects. They were also blinded to the findings of the other observer. To become familiar with the software, both radiologists applied the methodology in 10 adrenal glands before starting this study, and those data were not included in the analyses. Each radiologist measured the adrenal glands once. The measurement process took approximately 5 min per case.

Intraobserver agreement was assessed in 10 subjects (10 right adrenal glands and 10 left adrenal glands). Adrenal ADC measurements were repeated, by a single observer, two months after first measurements. Interobserver agreement was determined by comparing ADC values obtained by each radiologist for the right and left adrenal glands separately.

### Statistical analysis

Before statistical analysis, the measured values were checked for normal distribution with the Shapiro-Wilk test. Data were represented as means ± standard deviation (SD) or median and interquartile (25th-75th) range, as required. Univariate analysis for differentiating between the PreP and PostP was performed using the Student's *t*-test or Mann-Whitney test for covariates with and without normal distribution, respectively. Measurements made by the two reviewers were treated independently. For assessment of intraobserver and interobserver agreement, we calculated the intraclass correlation coefficient (ICC), which takes into account systematic and random errors^(18)^. Values of *p* < 0.05 were considered statistically significant. Statistical analysis was performed with the program R, version 2.15.0 (R Development Core Team; www.r-project.org) and the SPSS Statistics software package, version 19.0 (IBM Corp.; Armonk, NY, USA).

## RESULTS

### Apparent diffusion coefficient

**Figure 2:**
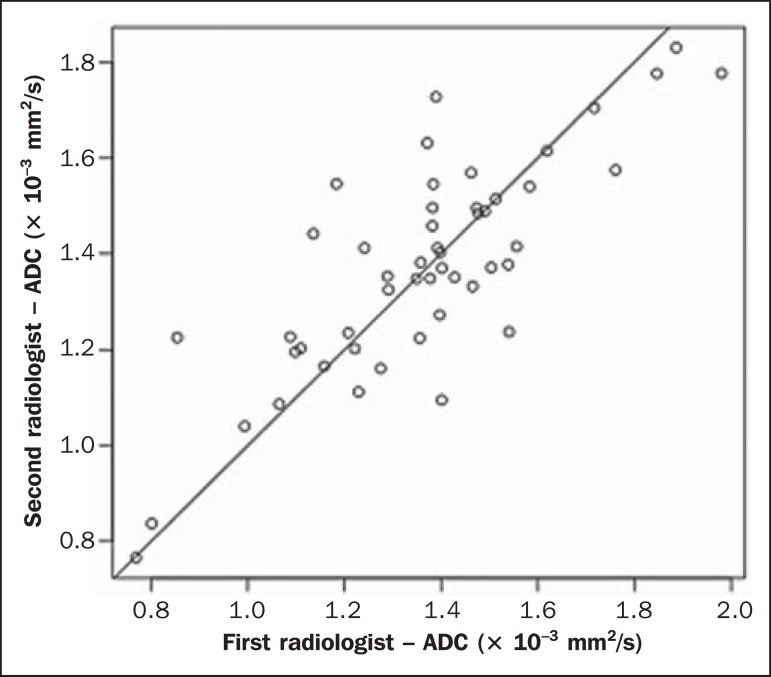
ADC values of the adrenal glands. Box-and-whisker plot of ADC measurements of the right and left adrenal glands (**A** and **B**, respectively). Boxes represent interquartile ranges. Whiskers represent ranges for all values. Horizontal lines within boxes are median values. Although there is a slight overlap of PreP and PostP group boxes, the means were significantly higher and different in the PostP group (*p* = 0.013 for the right side; *p* = 0.003 for the left side).

Mean ADC values for the right adrenal glands in the PreP and PostP groups were 1.44 × 10^-3^ mm^2^/s (SD, 0.23) and 1.23 × 10^-3^ mm^2^/s (SD, 0.21), respectively, compared with 1.58 × 10^-3^ mm^2^/s (SD, 0.22) and 1.32 × 10^-3^ mm^2^/s (SD, 0.23), respectively, for the left adrenal glands (Figure 2).

There was a significant difference in ADC values between the PreP and PostP groups, for the right adrenal glands (95% CI: 0.044 to 0.338; *p* = 0.013) and for the left adrenal glands (95% CI: 0.095 to 0.404; *p* = 0.003). There was no substantial difference between the ADC values for the right and left adrenal glands within the PreP group (95% CI: -0.3369188 to 0.0514484; *p* = 0.142) or within the PostP group (95% CI: -0.0916099 to 0.704502; *p* = 0.201). Differences between genders were also not significant, neither for the right adrenal glands (95% CI: -0.323 to 0.003; *p* = 0.104) nor for the left adrenal glands (95% CI: -0.365 to 0.0389; *p* = 0.116).

### Intraobserver and interobserver agreement

Intraobserver agreement for ADC measurements was high. The average ICC was 0.95 (95% CI: 0.82 to 0.98) for the right adrenal glands and 0.97 (95% CI: 0.91 to 0.99) for the left adrenal glands. For both sides together, the average ICC was 0.97 (95% CI: 0.93 to 0.99).

**Figure 3:**
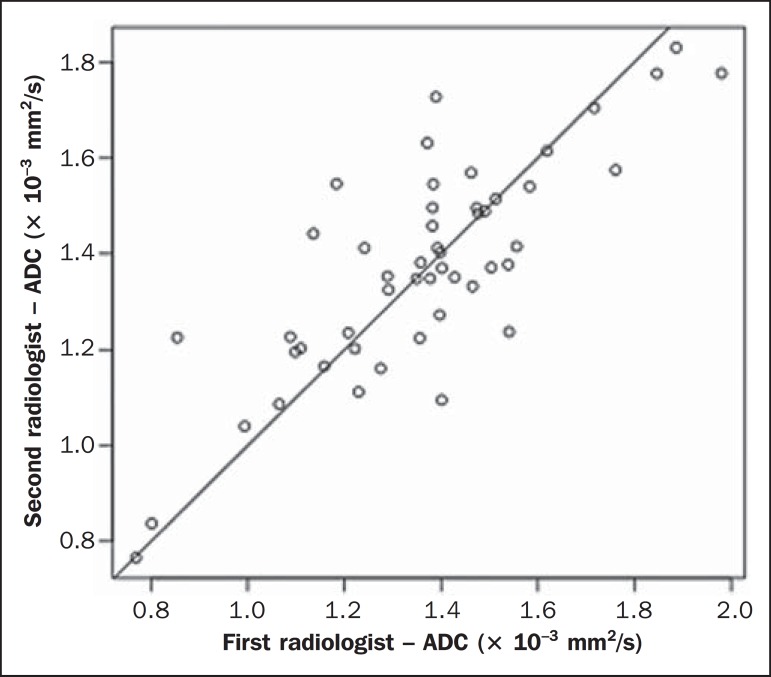
Interobserver agreement for ADC measurements of the adrenal glands. ICC for ADC values measured in the PreP and PostP groups. X axis, plotted measurements by the first radiologist; Y axis, plotted measurements performed by the second radiologist. ICC = 0.89 for the right and left sides (*p* < 0.001).

A high degree of interobserver agreement was found between the first and the second reviewers in terms of the ADC measurements, the average ICC ranging from 0.84 to 0.94 (Figure 3). In the sample as a whole, the average ICC was 0.84 (95% CI: 0.63 to 0.93) for the right adrenal glands, 0.94 (95% CI: 0.85 to 0.97) for the left adrenal glands, and 0.89 (95% CI: 0.81 to 0.94) for the left and right adrenal glands together; in the PreP group, the average ICC was 0.84 (95% CI: 0.46 to 0.95) for the right adrenal glands, 0.94 (95% CI: 0.59 to 0.99) for the left adrenal glands, and 0.88 (95% CI: 0.68 to 0.95) for the left and right adrenal glands together; and in the PostP group, the average ICC was 0.91 (95% CI: 0.76 to 0.98) for the right adrenal glands, 0.91 (95% CI: 0.76 to 0.97) for the left adrenal glands, and 0.87 (95% CI: 0.74 to 0.94) for the left and right adrenal glands together.

## DISCUSSION

Intravoxel incoherent motion (DW-MRI) images are quantified by ADC^(1)^. DW-MRI is a noninvasive, qualitative and quantitative technique that is very useful in differentiating among various pathological conditions. It has been used in the clinical routine, mainly in neuroimaging. With the development of echo planar imaging, together with fast and low artifact sequences, abdominal DW-MRI measurements became possible. After Müller et al.^(4)^ described the feasibility of performing DW-MRI and ADC measurements in abdominal exams, it came to be increasingly used as a biomarker in oncology^(19)^, as well as to evaluate focal and diffuse diseases^20)^. Additional roles of DW-MRI with ADC measurements include prediction of treatment outcomes^(21)^ and detection of lymph node involvement in cancer staging^22)^.

DW-MRI simultaneously provides information on diffusion and perfusion. When only high *b* values are applied, the influence of perfusion is largely cancelled out and the ADC value approximates true diffusion^(1)^. ADC values may be extrapolated using only two *b* values. However, sequences with multiple *b* values are more precise, with less contamination from perfusion^(23,24)^. The drawback of multiple *b* value sequences is temporal resolution. Generally for that, breath-hold techniques are not suitable and respiratory-gated sequences are required. In addition, some abdominal organs, such as the spleen and prostate, can show marked intrinsic water diffusion restriction, and high *b* values should be used for differentiation of normal and pathological tissues in abdominal MRI exams^(25)^. Therefore, in the present study, we used *b* values up to 1000 s/mm^2^.

Small lesions, especially those smaller than 1 cm, are very difficult to detect on ADC maps^(15)^. Normal adrenal glands show high signal intensity on DW-MRI, although their small size makes direct ADC evaluation difficult^(14)^. However, with the aforementioned approach, in which the DW-MRI sequence was fused with the ADC map, it was possible to obtain reliable ADC values. Fusion of DW-MRI with other sequences has been proposed for better evaluation of anatomic landmarks in lesions with irregular margins^(26)^, although not in small lesions or structures. The review of fusion images, evaluation of co-registration process, and referral to sequences other than DW-MRI improve confidence in the ADC values obtained^(27,28)^.

Our results show that the interobserver agreement for ADC measurements was almost perfect, unlike that described by Sandrasegaran et al.^(15)^, the difference likely being due to the methodology employed. We evaluated normal adrenal glands, whereas those authors studied large heterogeneous adrenal masses. In addition, we used a pixel-by-pixel method followed by calculation of the mean of all pixels, improved by an easier adrenal depiction in a sequence with better anatomical and contrast resolution which was fused with the ADC map. Although the lesions evaluated by Sandrasegaran et al.^(15)^ were larger than normal adrenal glands, the authors placed ROIs directly on the ADC maps.

The ADC values of some adrenal lesions have been assessed in previous studies^(11-15,29-32)^, leading to controversies. Some authors concluded that ADC values could not be used in order to differentiate between benign and malignant lesions^(12-15)^, although they could be used in order to identify benign pheochromocytomas^(12,14)^. However, others have shown that ADC might be a useful tool to evaluate indeterminate lesions^(15)^. Fractional anisotropy is another DW-MRI related parameter with the potential to differentiate between benign and malignant adrenal neoplasms^(11)^. Although the data were generated under different protocols and therefore are not truly comparable, when we analyze the ADC values found in previous studies, ADC values tend to be lower for malignant tumors than for normal adrenal glands. In fact, the ADC values of the normal adrenal glands evaluated in the present study are more similar to that reported for benign tumors, most of which were adenomas.

We found ADC values to be higher in the PreP subjects than in the PostP subjects, which may be due to adrenarche. The expansion of the zona reticularis is a hallmark of adrenarche^(33)^. In addition, it has been proposed that globular expansion of the mitochondrial cristae in the zona fasciculata correlates with steroidogenesis^(34)^. Both processes may lead to structural changes in the adrenal cortex resulting in decreased diffusion.

ADC values have also been calculated for normal abdominal organs^(8-10,35)^. However, to our knowledge, there have been no studies investigating ADC values of the normal adrenal glands. Our results support the reproducibility and feasibility of the method. After validation of this method, ADC values of normal adrenal glands may be used for comparison and follow-up of various abnormalities, particularly those with diffuse involvement of the adrenal glands, representing a new MRI parameter in this evaluation and adding to other newly developed techniques, such as the recently reported MR spectroscopy, for adrenal evaluation^(36-38)^. Nevertheless, DW-MRI is performed without breath holding, thus allowing examination of children, the severely ill, the elderly, or obese patients, who might be unable to cooperate during examination. The parameters of the sequence in the present study were in accordance with standard protocols used in literature^(5,39)^.

This study has some limitations. First, given the retrospective design, we included patients with normal MR exams and not healthy volunteers, although the exclusion of subjects at risk for adrenal or endocrine disorders, either acute or chronic, was probably sufficient to minimize the confounding factors related to adrenal pathology. Another limitation of this study is the small size of the sample. However, for our purposes, the group was sufficient to show that adrenal ADC measurement is possible and reproducible. And last, the spatial resolution provided by this technique, and MRI in general, precludes any conclusion regarding possible variations in ADC values due to histological differences among the two adrenal regions, the cortex, and the medulla. Rather, our results represent a mixture of pixels from these two histologically and functionally distinct zones.

## CONCLUSION

These preliminary results demonstrate the feasibility and reproducibility of ADC assessments of normal adrenal glands. We believe that these findings may add information for tissue characterization by MRI and may be used in the future to compare small adrenal lesions with normal adrenal glands. Our data also add information regarding the feasibility of ADC measurements of irregular and small structures.
